# Adrenomedullin Expression Is Associated With the Severity and Poor Prognosis of Interstitial Lung Disease in Dermatomyositis Patients

**DOI:** 10.3389/fimmu.2022.885142

**Published:** 2022-06-02

**Authors:** Lifang Ye, Yu Zuo, Fang Chen, Yuetong Xu, Puli Zhang, Hongxia Yang, Qinglin Peng, Guochun Wang, Xiaoming Shu

**Affiliations:** ^1^ Department of Rheumatology, Key Laboratory of Myositis, China–Japan Friendship Hospital, Beijing, China; ^2^ Peking Union Medical College, Chinese Academy of Medical Sciences, Beijing, China; ^3^ China-Japan Friendship School of Clinical Medicine, Peking University, Beijing, China

**Keywords:** adrenomedullin, interstitial lung disease, dermatomyositis, prognosis, disease severity

## Abstract

**Objective:**

To evaluate adrenomedullin mRNA levels in the peripheral blood mononuclear cells (PBMCs) of patients with dermatomyositis (DM) as well as their correlation with the severity of interstitial lung disease (ILD).

**Methods:**

A total of 41 DM patients and seven immune-mediated necrotizing myopathy (IMNM) patients were recruited, in addition to 21 healthy controls (HCs). The adrenomedullin mRNA levels in PBMCs were measured *via* quantitative reverse-transcription real-time polymerase chain reaction (qRT-PCR). The associations between adrenomedullin expression levels and major clinical, laboratory, pulmonary function parameters and the prognosis of patients with DM-related ILD (DM-ILD) were analyzed. Immunohistochemical analysis was performed on lung tissues of DM-ILD patients to determine adrenomedullin expression.

**Results:**

Adrenomedullin mRNA levels in PBMCs were significantly higher in DM patients than in IMNM patients and HCs (p = 0.022 and p<0.001, respectively). Among DM patients, the levels were significantly higher in those with rapidly progressive ILD (RP-ILD) than in those with chronic ILD (p = 0.002) or without ILD (p < 0.001). The adrenomedullin mRNA levels in DM-ILD were positively correlated with serum ferritin (r =0.507, p =0.002), lactate dehydrogenase (LDH) (r =0.350, p =0.045), and lung visual analog scale (VAS) (r=0.392, p=0.021) and were negatively correlated with pulmonary function test parameters, including predicted forced vital capacity (FVC)% (r = −0.523, p = 0.025), forced expiratory volume in 1 s (FEV1)% (r = -0.539, p = 0.020), and diffusing capacity of carbon monoxide (DLco)% (r = -0.495, p = 0.036). Immunohistochemical analysis of adrenomedullin confirmed higher expression in the alveolar epithelial cells and macrophages of DM patients with RP-ILD. Among the DM patients with ILD, the six decedents exhibited higher adrenomedullin levels than the 28 survivors (p = 0.042). The cumulative survival rate was significantly lower (62.5% *vs*. 100%, P = 0.005) in patients with an adrenomedullin level > 0.053 than in those with a level <0.053.

**Conclusions:**

Adrenomedullin levels are upregulated in DM patients with RP-ILD and are associated with ILD severity and poor prognosis. Adrenomedullin may be a potential prognostic biomarker in DM patients with ILD, although need further investigation.

## Introduction

Dermatomyositis (DM) encompasses a group of heterogeneous autoimmune conditions that affect not only the muscles and skin, but also several other organs including the lungs and heart (1, 2). Interstitial lung disease (ILD) is considered the most common and serious complication of DM and is usually resistant to high-dose glucocorticoids or other immunosuppressive therapy, contributing the morbidity and mortality of DM patients. ILD is difficult to detect in the early stages of disease. Some patients with DM-ILD develop rapidly progressive ILD (RP-ILD) within three months of onset. Without effective treatment, only 40-45% of these patients survive after 6-months (3–6). Although several biomarkers, such as ferritin (7, 8), IL-18 (9), and Krebs von Nest Lungen-6 (KL-6) have been used as indexes for inflammatory activity within the lungs (10, 11), the exact mechanism of DM-ILD pathogenesis remains unclear.

Adrenomedullin is a bioactive peptide composed of 52 amino acids, originally found in the acid extract of human pheochromocytoma tissue (12). Studies have shown that it is not only produced in the normal adrenal medulla but is also widely distributed in various tissues and cell types, including alveolar macrophages, bronchoalveolar epithelial cells, and lung endothelial cells (13–15). Adrenomedullin participates in various pathological and physiological processes, including in inflammatory pathways, and regulates angiogenesis and lung tissue repair (16). Studies have confirmed that plasma adrenomedullin levels are increased in certain connective tissue diseases. For example, the increased expression of plasma and cellular adrenomedullin has been widely associated with disease activity in rheumatic diseases, including systemic sclerosis, systemic lupus erythematosus, and rheumatoid arthritis. These studies also revealed that adrenomedullin is involved in the pathogenesis of the above-described conditions (16–20). It was recently reported that the expression of plasma adrenomedullin increases in chronic obstructive pulmonary disease (COPD), which may reflect the severity of disease and serve as an independent predictor of prognosis (21). However, whether adrenomedullin levels are elevated in DM patients as well as the relationship between adrenomedullin levels and ILD severity remains unknown.

Here, we investigated adrenomedullin expression levels in the PBMCs of patients with DM and assessed the association of adrenomedullin with clinical characteristics, focusing on ILD and prognosis.

## Materials and Methods

### Patients

**Figure 1:**
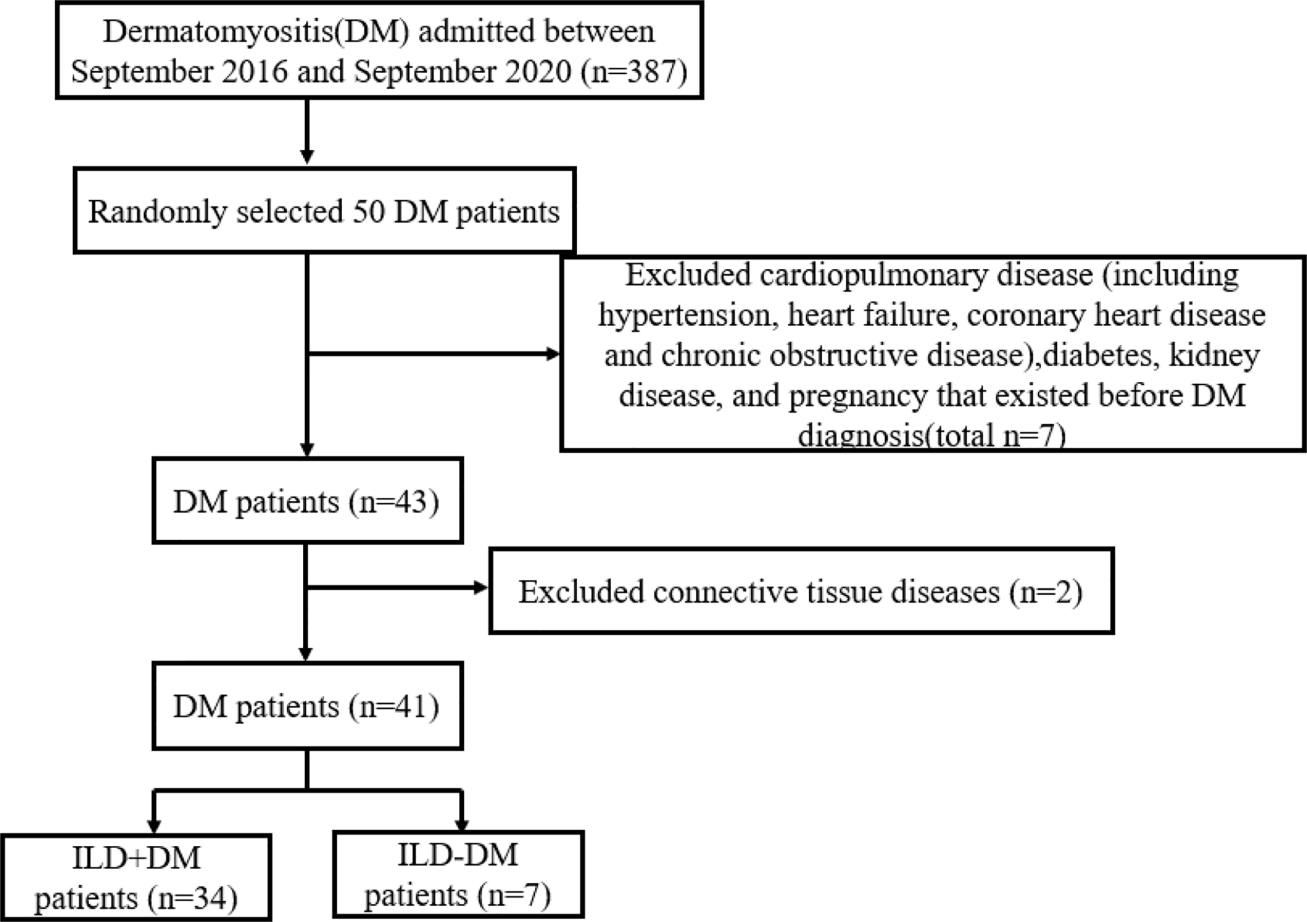
Selection flowchart.

A total of 41 DM and seven IMNM patients admitted to the China-Japan Friendship Hospital between September 2016 and September 2020 were enrolled in our study. All patients were diagnosed according to the 2017 ACR/EULAR classification criteria for IIMs (22), IMNM was classified by ENMC IMNM classification criteria (23). Patients complicated with other connective tissue diseases and those aged <18 years were excluded from the current study. We also excluded patients with following conditions which include cardiopulmonary disease (including hypertension, heart failure, coronary heart disease and chronic obstructive disease), diabetes, kidney disease, and pregnancy, because it has been reported that the levels of adrenomedullin were elevated in patients with these conditions (15) before study. The selection process for DM patients was shown in **Figure 1**. In addition, 21 sex- and age- matched healthy controls (HCs) from the Physical Examination Center of the China-Japan Friendship Hospital were included. Patients’ medical history information was obtained from the hospital’s electronic information system, including age, sex, clinical manifestations, laboratory data, and lung function tests. Physician global assessment (PGA), which was recorded on a continuous 10 cm visual analog scale (VAS), was used to assess disease activity of patients with DM. The scale provides a comprehensive score for the whole body, including the joints, heart, lungs, gastrointestinal system, skin, and muscle organs or systems (24). All patients in the current study were followed up with for at least 12 months until July 2021. Each participant gave written informed consent before enrollment, and the study was approved by the Research Review Committee and Ethics Review Committee of the China-Japan Friendship Hospital.

### Classification of ILD

ILD diagnosis was established by the high-resolution computed tomography (HRCT) (25). DM patients with ILD were divided into two clinical subgroups, RP-ILD and chronic ILD. RP-ILD is defined as rapidly progressing ILD with severe dyspnea symptoms and new interstitial abnormalities on HRCT within 3 months (25). The diagnosis of chronic ILD is based on asymptomatic, slowly progressive ILD or non-rapidly progressive ILD imaging for more than 3 months (26).

### Measurement of Adrenomedullin

Peripheral blood mononuclear cells (PBMCs) were separated from 6 ml of peripheral blood samples of DM, IMNM, and HCs *via* Histopaque (1.077g/mL, Sigma-aldrich, St. Louis, USA) density gradient centrifugation and stored in liquid nitrogen. The rapid RNA extraction kit (Yishan, Shanghai, China) was used to extract total RNA from PBMCs, and RNA concentration was quantified using a NanoDrop 2000 spectrophotometer (Thermo Scientific, Waltham, MA, USA). Total RNA was reverse transcribed into cDNA using the PrimeScriptTM RT reagent (Takara, Otsu,Japan). To compare the mRNA levels of adrenomedullin (forward primer: 5’-TTGTCCTCCCCTATTTTAAGACG -3’, reverse primer: 5’-CTTCCACACAGGAGGTAATCAGTC-3’), qRT-PCR was performed using an ABI 7500 sequence detection system (Applied Biosystems, Foster City, CA) with SYBR Green Master Mix (Qiagen, Hilden, Germany). The cycling conditions were as follows: 95°C for 2 min, followed by 40 cycles of 95°C for 5 s and 60°C for 30 s. Stability analysis and identification of candidate internal reference genes are listed in **Supplementary material**. Geometric mean of RBS18 and GAPDH was used as the reference genes for gene expression analysis. Each sample was measured in triplicate. The 2^-ΔCt^ method was used to calculate relative expression levels of RNA normalized to an endogenous control.

### Immunohistochemistry

Lung tissue sections were obtained *via* percutaneous lung biopsy. 10% formalin was used to fix the tissues. The fixed tissues were embedded in paraffin (6 µm thickness), deparaffinized, and preheated for 30 min in epitope retrieval solution (Citric acid Retrieval Solution, Aladdin, Shanghai, China). The tissue sections were incubated with rabbit anti-adrenomedullin polyclonal antibody (1:500 dilution; Proteintech, Wuhan, China) overnight at 4°C, and then incubated with a goat anti-rabbit IgG secondary antibody (Gene Tech Shanghai Company Limited, Shanghai, China) for 30 min at room temperature. Peroxidase activity was determined using 3,3’-Diaminobenzidine (Gene Tech Shanghai Company Limited). The tissues were counterstained with hematoxylin.

### Statistical Analysis

Normally distributed data were expressed as the mean ± standard deviation (SD) and compared by a t-test. Non-normally distributed data were expressed as median [with interquartile range (IQR)] and were compared by the Mann-Whitney U test. Spearman’s correlation analysis was used to test correlations. Fisher’s exact test was performed to calculate categorical data. When predicting patient prognosis, the best predicted cut-off points were calculated using receiver operating characteristic (ROC) analysis. The Kaplan-Meier test was used to calculate survival rates. Differences of p < 0.05 were considered statistically significant. Statistical analysis was carried out using SPSS 25.0 and GraphPad Prism 8.0.

## Results

### Comparison of Baseline Clinical Features Between DM Patients With or Without RP-ILD

There were 41 patients with DM recruited in the current study. Among these, 30 were women. The mean onset age was 52.0 years, and the median disease duration was 6.0 months. There were 34 patients (82.9%) with ILD, while 13 (31.7%) were classified as having RP-ILD. Clinical features, laboratory data, and pulmonary function test parameters are described in **Table 1**. The onset age in DM patients with RP-ILD was significantly higher than in those without ILD. Further, the frequency of mechanic’s hands and skin ulcers was significantly higher in DM patients with RP-ILD than in those with chronic ILD. The frequency of anti-MDA5 positivity was significantly different between DM patients with RP-ILD and those without ILD. Ferritin levels, which indicate ILD disease activity, were significantly elevated in patients with RP-ILD than in those without it. Moreover, CEA levels were obviously higher in patients with RP-ILD than in those without ILD. The FVC% and FEV1% indicated more severe pulmonary involvement in DM patients with RP-ILD. All DM patients received corticosteroids plus other immunosuppressive agents, with 24.3% receiving triple therapy including corticosteroids, immunosuppressants (cyclosporine or tacrolimus), and intravenous cyclophosphamide. Intravenous immunoglobulin (IVIG) therapy was more frequently used in patients with RP-ILD than in those with chronic ILD (**Table 1**).

**Table 1:** Clinical and laboratory characteristics of DM patients with *vs* without RP-ILD.

Characteristics	DM with RP-ILD (n=13)	DM with chronic ILD (n=21)	DM without ILD (n=7)
Female gender, no. (%)	9 (69.2%)	15(71.4%)	6(85.7%)
Onset age (years, mean ± SD)	55.0 ± 5.3	51.0 ± 11.3	30.7 ± 11.8***
Disease duration [months, median (IQR)]	4.0 (1.5-7.5)	8.0(4.0-19.0)	2.0(1.3-9.0)
Clinical features, no. (%)			
Muscle weakness	5 (38.4%)	9 (42.8%)	6(85.7%)
Heliotrope rash	8 (61.5%)	10 (47.6%)	6(85.7%)
Gottron papules	9 (69.2%)	8 (38.0%)	2(28.5%)
Mechanic’s hands	10 (76.9%)	4 (19.0%)**	2(28.5%)
V sign, no. (%)	6 (46.1%)	8 (38.0%)	5(71.4%)
Skin ulcers	3 (23.0%)	0 (0)*	0(0)
Arthritis/arthralgia	5 (38.4%)	6 (28.5%)	1(14.2%)
Dysphagia	2 (15.3%)	2 (9.5%)	2(28.5%)
Laboratory examinations			
Anti-ARS	0 (0)	5( 23.8%)	1(14.2%)
Anti-MDA-5	12 (92.3%)	13 (61.9%)	0(0)***
CK, IU/l	39.0 (18.0-104.5)	49.0 (32.0-149.0)	311.0(40.0-1553.0)
LDH, IU/l^a^	332.5 (279.8-442.0)	297.0 (226.0-447.5)	308.0(190.0-448.0)
AST, IU/l	38.0 (17.5-51.0)	21.0 (17.0-35.5)	49.0(15.0-81.0)
ALT, IU/l	46.0 (25.5-84.0)	28.0 (16.5-73.5)	38.0(19.0-147.0)
CRP, mg/dl^a^	0.46 (0.25-0.71)	0.62 (0.21-1.35)	0.17(0.14-0.46)
ESR, mm/H	33.0 (15.5-42.0)	8.0 (6.0-38.0)	7.0(3.0-16.0)
Ferritin, ng/ml^b^	1011 (477.6-1849)	367.2 (70.2-875.9)*	172.4(32.6-565.2)**
CEA, ng/ml^c^	5.3 (2.6-12.4)	2.5 (1.3-4.9)	1.4(0.8-1.6)**
CYFRA211, ng/ml^d^	4.7 (3.2-6.3)	3.3 (2.0-6.7)	2.6(1.8-22.5)
Pulmonary function test			
FVC (%)^e^	74.24 ± 22.01	93.43 ± 15.66	105.70 ± 2.62**
FEV1 (%)^e^	70.38 ± 19.44	83.84 ± 11.50	111.10 ± 6.36**
DLCO (%)^e^	63.16 ± 20.34	73.71 ± 12.84	93.97 ± 24.33
Treatment			
Corticosteroid alone, n (%)	0	3 (14.2%)	0
Corticosteroid+ immunosuppressant, n (%)	13 (100%)	20 (95.2%)	6(85.7%)
cyclosporine, IVCY, n (%)	10 (76.9%),2(15.3%)	12 (57.1%), 3(14.2%)	0, 2(28.5%)
Triple treatment, n (%)	4 (30.7%)	5 (23.8%)	1(14.2%)
IVIG, n (%)	6 (46.1%)	2 (9.5%)*	1(14.2%)

DM, dermatomyositis; ILD, interstitial lung disease; RP‐ILD, rapidly progressive ILD; CK, creatine kinase; LDH, Lactate dehydrogenase; AST, aspartate aminotransferase; ALT, alanine aminotransferase; CRP, C-reactive protein; ESR, erythrocyte sedimentation rate; CEA, carcinoembryonic antigen; CYFRA211:Cytokeratin 19 fragment; FVC, forced vital capacity; FEV1, forced expiratory volume in 1s; DLco, diffusing capacity of carbon monoxide; IVCY, intravenous cyclophosphamide; Triple treatment, corticosteroid, immunosuppressants (ciclosporin or tacrolimus) and intravenous cyclophosphamide; IVIG, intravenous immunoglobulin. a,b,c,d,e Data were available for 40, 39,38,31 and 21 patients, respectively.*P < 0.05,^∗∗^P < 0.01, and ^∗∗∗^P < 0.001.

### Adrenomedullin mRNA Expression Levels in PBMCs Were Markedly Increased in DM Patients With RP-ILD

**Figure 2:**
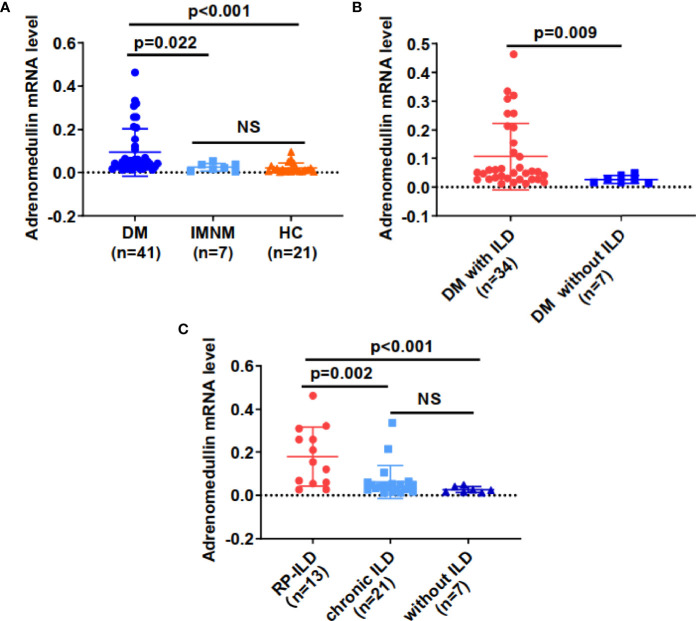
Adrenomedullin mRNA levels from PBMCs were elevated in DM patients with RP-ILD. **(A)** Adrenomedullin mRNA levels in DM patients were significantly higher than those in IMNM patients and HCs. **(B)** Adrenomedullin mRNA levels in DM patients with ILD and those without ILD. **(C)** The adrenomedullin mRNA levels in DM patients with RP-ILD, chronic ILD, and those without ILD. DM, dermatomyositis; IMNM, immune-mediated necrotizing myopathy; HC, healthy control; ILD, interstitial lung disease; RP-ILD, rapidly progressive interstitial lung disease. NS indicates no significant difference. Differences between the levels were expressed as relative expression *via* the 2^-ΔCt^ method. Data were expressed as the mean ± standard deviation (SD).

To elucidate the association between adrenomedullin and DM, we first compared adrenomedullin mRNA levels among PBMCs from 41 patients with DM, 7 patients with IMNM, and 21 healthy controls (HCs) (**Figure 2A**). No significant age or sex difference was observed among the three groups (data not shown). The median adrenomedullin mRNA level in patients with DM was 0.047 (0.026-0.112), which was significantly higher than that in IMNM patients (0.020 [0.004-0.036], p = 0.022) and HCs (0.011 [0.004-0.025], p < 0.001) (**Figure 2A**). However, no significant difference in adrenomedullin expression was observed between IMNM patients and HCs. We then analyzed the relationship between adrenomedullin expression and clinical characteristics. We observed that adrenomedullin expression was significantly higher in patients with ILD than in those without (p = 0.009) (**Figure 2B**). The former group was further divided into patients with RP-ILD or chronic ILD. The adrenomedullin mRNA levels in patients with RP-ILD were significantly higher than in those with chronic ILD and without ILD (p = 0.002 and <0.001, respectively) (**Figure 2C**). No statistical difference was observed in the expression of adrenomedullin mRNA between patients with chronic ILD and those without ILD. These results indicate that adrenomedullin mRNA levels are closely related to ILD, especially RP-ILD.

### Elevated Adrenomedullin mRNA Expression Levels in PBMCs Were Correlated With the Severity of Lung Involvement in DM Patients With ILD

**Figure 3:**
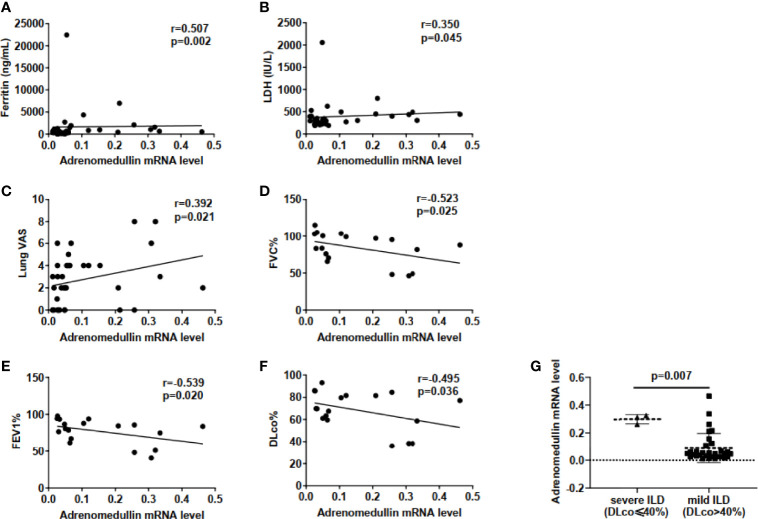
Elevated adrenomedullin mRNA levels in PBMCs were correlated with the severity of lung involvement in DM patients with ILD. **(A)** Adrenomedullin mRNA Levels were positively correlated with ferritin levels in DM-ILD. **(B)** Adrenomedullin mRNA Levels were positively correlated with LDH levels in DM-ILD. **(C)** Correlation between adrenomedullin mRNA levels and lung VAS in DM-ILD. **(D)** Adrenomedullin mRNA levels were negatively correlated with FVC% in DM-ILD. **(E)** Adrenomedullin mRNA levels were negatively correlated with FEV1% in DM-ILD. **(F)** Adrenomedullin mRNA levels were negatively correlated with DLco% in DM-ILD. **(G)** Adrenomedullin mRNA expression levels in patients with severe ILD (n = 3) and mild-moderate ILD (n = 15). DM, dermatomyositis; ILD, interstitial lung disease; LDH, lactate dehydrogenase; VAS, visual analogue scale; FVC, forced vital capacity; FEV1, forced expiratory volume in 1s; DLco, carbon monoxide diffusion capacity.

We investigated the association among adrenomedullin mRNA levels in PBMCs and various clinical parameters in 34 DM patients with ILD. The adrenomedullin mRNA levels were positively associated with serum ferritin (r = 0.507, p = 0.002) and lactate dehydrogenase (LDH) (r = 0.350, p = 0.045) (**Figures 3A, B**). No association was found between adrenomedullin levels and C-reactive protein (CRP), creatine kinase (CK), or erythrocyte sedimentation rate (ESR) (p-values > 0.05). Additionally, lung VAS scores were evaluated when blood samples were collected. We found that adrenomedullin mRNA levels were positively associated with the lung VAS score (r = 0.392, p = 0.021) (**Figure 3C**). To test the association between adrenomedullin mRNA levels in PBMCs and the severity of lung involvement in DM patients with ILD, we analyzed the correlation between adrenomedullin mRNA levels and pulmonary function test (PFT) parameters, including FVC%, FEV1%, and DLco%. Although only 18 patients underwent pulmonary function tests, the results showed that FVC%, FEV1%, and DLco% were negatively associated with adrenomedullin mRNA levels (r = −0.523, -0.539, and -0.495; p = 0.025, 0.020, and 0.036, respectively) (**Figures 3D–F**). DLco%≤40% was defined as severe ILD, and this group of DM patients had significantly higher adrenomedullin mRNA levels than patients with mild-moderate ILD (DLco%>40%) (**Figure 3G**). These results reveal that DM patients with ILD have higher adrenomedullin mRNA levels and present with more severe pulmonary symptoms.

### Immunohistochemical Analysis for Adrenomedullin Expression in the Lung Tissues

**Figure 4:**
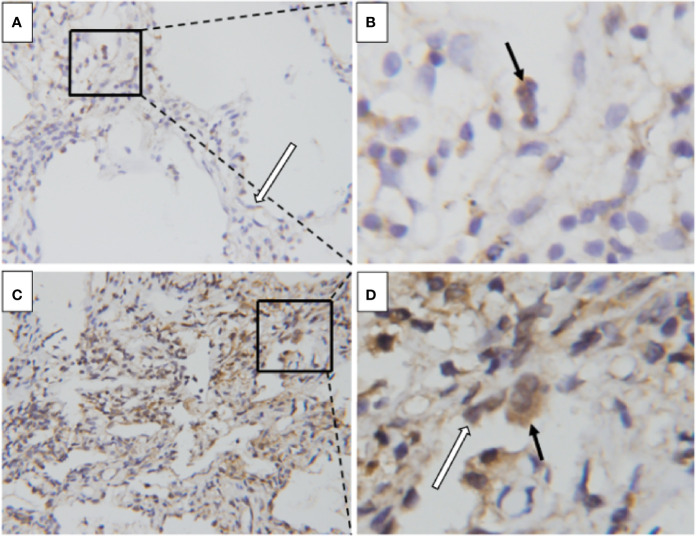
Enhanced adrenomedullin expression in the lung tissue of DM patients with RP-ILD. **(A, B)** The lung of a DM patient with chronic ILD. Positive adrenomedullin staining was detected in macrophages (black arrowhead) and alveolar epithelial cells (white arrowhead). **(C, D)** The lung of a DM patient with RP-ILD. Positive adrenomedullin staining was detected in macrophages (black arrowhead) and alveolar epithelial cells (white arrowhead). Scale bar = 50 μm. DM, dermatomyositis; ILD, interstitial lung disease; RP-ILD, rapidly progressive ILD.

To investigate the cellular location and the source of adrenomedullin in the lungs, we performed immunohistochemical staining for adrenomedullin from two patients with DM patients with ILD. Sections from DM patients with chronic ILD exhibited weak staining for adrenomedullin in macrophages and alveolar epithelial cells (**Figures 4A, B**), whereas those from DM patients with RP-ILD were strongly positive for adrenomedullin in macrophages and alveolar epithelial cells (**Figures 4C, D**).

### Survival Analysis Based on Adrenomedullin mRNA Levels in DM Patients With ILD

**Figure 5:**
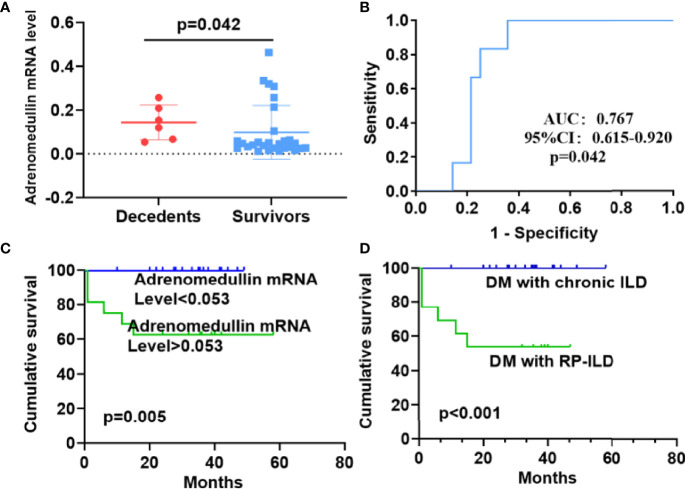
Prognostic value of adrenomedullin in DM patients with ILD and survival analysis. **(A)** The adrenomedullin mRNA levels in PBMCs were higher in decedents than survivors among DM patients with ILD. **(B)** Receiver operating characteristic curve analyses to predict the mortality of DM patients with ILD. **(C)** The cumulative survival rate was significantly lower in the group with adrenomedullin mRNA levels >0.053 than in those with adrenomedullin mRNA levels <0.053 (62.5% *vs* 100%, log-rank test, P = 0.005). **(D)** Kaplan–Meier curves showed that the cumulative survival rate was significantly lower in DM patients with RP-ILD than in those without RP-ILD (53.8% *vs* 100%, log-rank test, P < 0.001). DM, dermatomyositis; ILD, interstitial lung disease; RP‐ILD, rapidly progressive ILD.

As adrenomedullin mRNA levels were elevated in DM patients with RP-ILD and involved in more severe pulmonary symptoms, we investigated their relationship with patient survival. Adrenomedullin mRNA levels in PBMCs from DM patients with ILD were significantly higher in decedents than those in survivors (0.137 [0.064-0.221] *vs* 0.047 [0.026-0.094], p = 0.042) (**Figure 5A**). To accurately distinguish between decedents and survivors, we identified the cut-off value for adrenomedullin mRNA levels in PBMCs. In the ROC curve analysis, the highest AUC of adrenomedullin was 0.767, with a cut-off value of 0.053 (**Figure 5B**). Similarly, we calculated the optimal cut-off values for ferritin and LDH to distinguish between decedents and survivors as well. The AUCs for ferritin was 0.778, close to that of adrenomedullin, suggesting comparable values of adrenomedullin and ferritin in predicting decedents in DM-ILD. But we did not observe similar value of LDH in our samples. Based on this cut-off value (sensitivity, 100%; specificity, 64.2%), the patients were divided into high or low adrenomedullin level groups. The 1‐year cumulative survival rate of the patient group with adrenomedullin mRNA levels > 0.053 was 62.5%, while the patient group with adrenomedullin mRNA levels <0.053 had a survival rate of 100% (p= 0.005) (**Figure 5C**). Finally, Kaplan–Meier survival curves were performed for DM patients based on RP-ILD status. The 1‐year cumulative survival rate for patients with chronic ILD was 100%, whereas that for patients with DM and RP-ILD was 53.8%. RP-ILD status was closely corrected with a higher risk of mortality (p <0.001) (**Figure 5D**).

## Discussion

In this study, we revealed that adrenomedullin mRNA levels are obviously increased in DM patients, especially in those with RP-ILD. Adrenomedullin expression was elevated in the lung tissues specimens of patients with RP-ILD. Further, increased adrenomedullin levels were associated with clinical indicators associated with ILD and poor prognosis. This is the first study to determine the clinical significance of PBMC adrenomedullin in DM.

A previous study showed that plasma and joint tissue adrenomedullin concentrations were higher in patients with rheumatoid arthritis (RA) than in those with osteoarthritis and increased with RA disease activity (19). In addition, plasma adrenomedullin and CRP levels were positively correlated. Adrenomedullin may participate in the regulation of inflammatory processes in RA (19). Additional studies identified constitutive adrenomedullin expression in PBMCs from patients with RA and suggested multiple biological roles (20). Plasma adrenomedullin levels were obviously elevated in patients with systemic lupus erythematosus (SLE) compared to those in healthy controls, and a positive correlation was found between plasma adrenomedullin levels and SLEDAI-2K, which may serve as a potential indicator of disease activity (17). In the current study, we found that adrenomedullin mRNA levels from PBMC were higher in DM patients than in HCs or IMNM patients. Previous studies have shown that proinflammatory cytokines, such as interleukin-6 (IL-6), tumor necrosis factor (TNF)-α, and IL-1, stimulate adrenomedullin expression in smooth muscle, endothelial cells, and macrophages (27, 28). These pro-inflammatory cytokines play a significant role in mediating the inflammatory pathogenesis of DM (29, 30). Further, they may stimulate adrenomedullin production in various cell types in DM. Mononuclear macrophages are an important cell population responsible for elevated plasma adrenomedullin (31). Monocytes are among the key immune cell types involved in DM immunopathogenesis (2). These mechanisms may underpin the elevated levels of adrenomedullin in DM patients.

**Figure 6:**
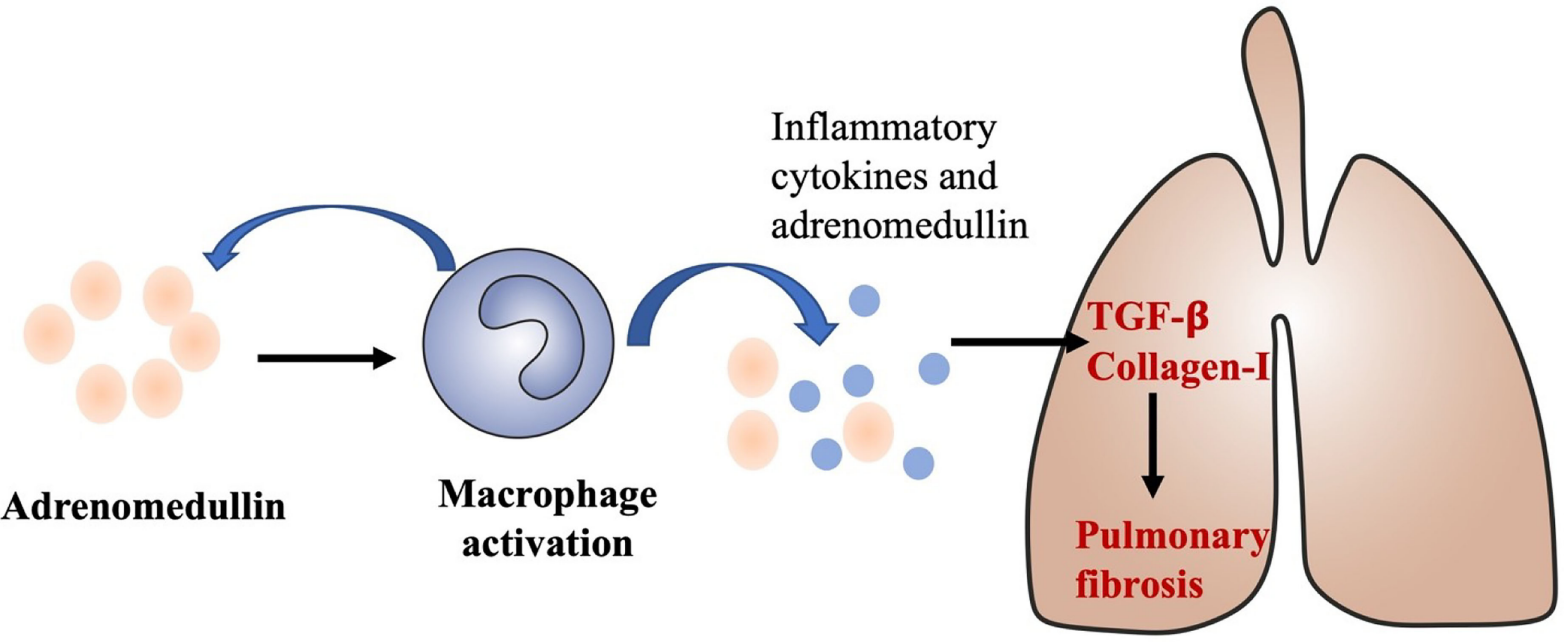
A proposed role of adrenomedullin in the develop and progress of ILD in DM. Adrenomedullin stimulates macrophage activations and activated macrophage could also secrete adrenomedullin and other inflammatory cytokines, type I interferon, promote the production of TGF-β, and collagen-I which could contribute to the develop and progress of ILD in DM.

Our study shows that adrenomedullin mRNA levels are correlated with disease severity in DM patients with ILD, upregulated in both the PBMCs and tissues. Moreover, immunostaining of ILD specimens revealed that adrenomedullin was expressed not only in alveolar epithelial cells, but also in macrophages, suggesting that adrenomedullin may be involved in inflammatory processes in DM patients with ILD. RP-ILD is characterized by aberrant inflammation (6–10). In DM patients with RP-ILD, macrophage activation is known to be involved in pathogenic processes (7, 32). The number of CD163-positive macrophages in the alveolar space was significantly increased in patients with RP-ILD compared with patients with chronic ILD (32). Studies have shown that serum ferritin level are a key biomarker of macrophage activation and can indicate the severity of RP-ILD in DM patients (9). Macrophages can produce adrenomedullin, which is induced by inflammatory cytokines (28). Inflammation can damage lung cells in RP-ILD leading to the release of LDH into blood (11). We found that PBMC adrenomedullin levels were positively correlated with the above RP-ILD-related markers. Higher levels of adrenomedullin may reflect disease progression in RP-ILD. Therefore, adrenomedullin may act as a reflection of the inflammatory state and participate in the pathogenesis of DM with RP-ILD (**Figure 6**).

We found that PBMC adrenomedullin levels in DM patients with ILD associated with pulmonary function test results, higher adrenomedullin mRNA levels correlated with worse lung function in DM patients with ILD, which may help assess the severity of ILD (33, 34). In addition, deterioration of lung function, especially forced vital capacity (FVC) and DLco, is known to indicate disease progression and to be corelated with prognosis in DM patients with ILD (34). Therefore, PBMC adrenomedullin levels can be indicative of disease severity and prognosis in these patients. Herein, we found that the cumulative survival rate of the PBMC adrenomedullin levels > 0.053 group was obviously decreased. These data reveal that PBMC adrenomedullin levels may act as a prognostic factor. Therefore, highly increased adrenomedullin mRNA levels require more active attention and management in clinical practice.

The current study had several limitations. First, the sample size was relatively small and was a retrospective study, which affects further analysis and statistical power. In order to prove that the observed effect is not due to chance, a larger sample size is usually required. Second, the serum adrenomedullin levels of patients were not determined. As the current study was retrospective and carried out at a single center, large-scale prospective observational studies will help validate the prognostic significance of adrenomedullin. Third, we only did immunohistochemical staining in one patient with RP-ILD and C-ILD, respectively, which could not acquire the statistical analysis. At last, the exclusion of some diseases, such as diabetes, limit its generalizability and clinical value to some extent, however, our results still suggest a correlation with severity and poor prognosis of interstitial lung disease.

In conclusion, the current study preliminarily shows the upregulation of adrenomedullin levels in PBMCs from DM patients, especially those with RP-ILD. Further, we evaluated the prognostic value of adrenomedullin expression in patients with DM-ILD. Our findings provide novel insights into the pathogenesis of DM. Further research is needed to elucidate the working mechanism of adrenomedullin in DM.

## Data Availability Statement

The original contributions presented in the study are included in the article/**Supplementary Material**. Further inquiries can be directed to the corresponding authors.

## Ethics Statement

The studies involving human participants were reviewed and approved by the Ethics Committee of China–Japan Friendship Hospital. The patients/participants provided their written informed consent to participate in this study. Written informed consent was obtained from the individual(s) for the publication of any potentially identifiable images or data included in this article.

## Author Contributions

LY, YZ, FC, YX, PZ, and HY participated in collecting and analyzing the data. LY wrote the manuscript. QP, GW, and XS supervised the manuscript. All authors contributed to the article and approved the submitted version.

## Funding

This work was supported by the Elite Medical Professionals project of China-Japan Friendship Hospital (NO. ZRJY2021-GG14), the Youth Program of the National Natural Science Foundation of China (grant numbers 81401363, 81601367), National Natural Science Foundation of China (grant number 81971531), Capital Health Research and Development of Special Programs (grant number 2014–4-4062), and The Fundamental Research Funds for the Central Universities (grant number 3332020074).

## Conflict of Interest

The authors declare that the research was conducted in the absence of any commercial or financial relationships that could be construed as a potential conflict of interest.

## Publisher’s Note

All claims expressed in this article are solely those of the authors and do not necessarily represent those of their affiliated organizations, or those of the publisher, the editors and the reviewers. Any product that may be evaluated in this article, or claim that may be made by its manufacturer, is not guaranteed or endorsed by the publisher.
