# Supplementary material for: Adrenomedullin Expression Is Associated With the Severity and Poor Prognosis of Interstitial Lung Disease in Dermatomyositis Patients
Source: Front Immunol. 2022 Jun 2;13:885142. doi: 10.3389/fimmu.2022.885142 (PMC9200949; doi:10.3389/fimmu.2022.885142)
Supplement: Supplementary file 1 [file DataSheet_1.pdf]

Supplementary Material

Supplementary Results

1 Expression pattern of candidate reference genes in PBMC

In order to identify appropriate reference gene for mRNA quantification in PBMCs, four candidate reference genes (ACTB, UBE2D2, RPS18, GAPDH), chosen from the literature (1-2), were examined in human PBMCs. Ten patients with dermatomyositis (DM) and 8 age and gender-matched healthy controls (HCs) were included in the study. Among the candidate reference genes, a wide spectrum of Ct values ranging from 16.33 to 29.38 was observed. Among all, ACTB was the most abundant candidate with a median Ct of 18.71. The median Ct values of others are shown in supplementary table 1.

Supplementary Table 1 Descriptive statistical values of Cycle of quantification of reference genes in 18 tested samples

Rank	Gene	Minimum	Max	Average	Median	SD
1	ACTB	16.33	21.41	18.71	18.71	1.27
2	UBE2D2	22.08	29.38	24.51	23.93	1.81
3	RPS18	18.91	22.84	20.10	19.50	1.18
4	GAPDH	19.22	22.59	20.73	20.54	1.04

2 Expression of candidate genes in patients and healthy controls

None of the reference genes were affected by the disease state since observed Ct values did not show any significant difference between DM patients and HCs (Supplementary Figure 1). In addition, the result of t test with P>0.05 for all reference genes indicated that the tested populations of patients and controls have equal variance. This is an essential step before evaluating the stability of reference genes as the algorithms used assume there is no difference in expression pattern of candidate genes between experimental groups.

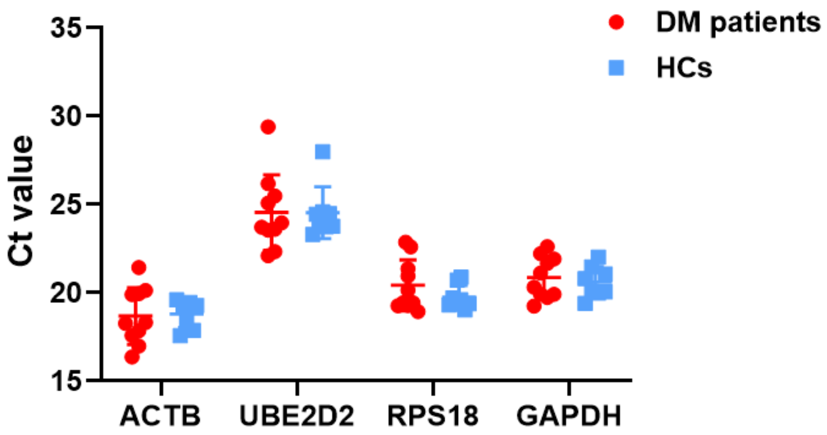

Supplementary Figure 1 Ct values of candidate genes in tested samples. No differences were found between the DM patients and HCs (P>0.05). Ct: Cycle of threshold

To visualize expression variation of reference genes in these samples, raw Ct values were plotted. As shown in supplementary figure 2, the highest variation belonged to ACTB and UBE2D2, therefore being the two least stable reference genes (Supplementary Figure 2).

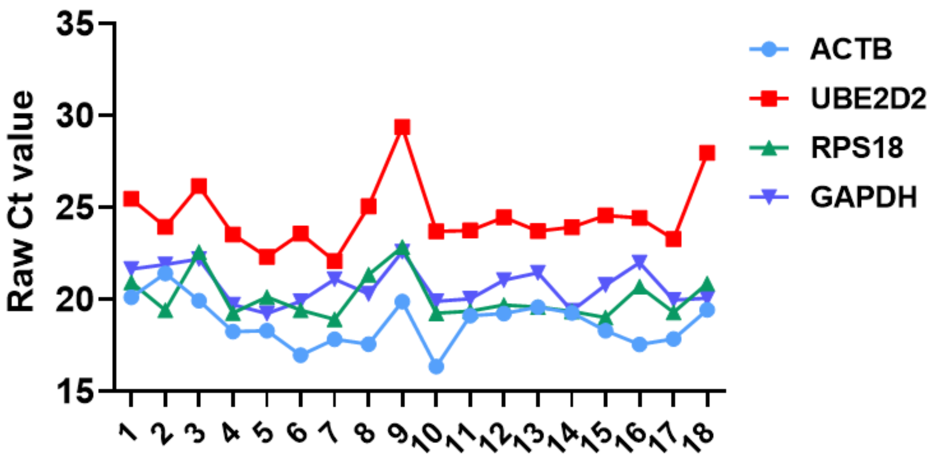

Supplementary Figure 2 The variation in expression level of 4 reference genes investigated in PBMC samples of 18 participants in this study. The expression level was estimated using qRT-PCR (based on duplicate readings). qRT-PCR: quantitative reverse-transcription polymerase chain reaction PCR.

3 Analysis of candidate reference genes using Silver's method, geNorm, BestKeeper and NormFinder

All the employed software programs assign a relative stability value to each candidate gene which is inversely correlated to its stability. The results of expression stability values are shown in Supplementary Table 2. All the software programs used specified a comprehensive highest stability for RPS18 and a lowest stability for UBE2D2. The second mostly stable reference gene was GAPDH. Therefore, to reach a conclusion, comprehensive gene-stability value was also calculated for each gene (Supplementary Table 2).

Supplementary Table 2 Expression stability of reference genes across 18 tested samples.

Rank	Gene	NormFinder	geNorm	BesteKeper	Delta Ct	Comprehensive
1	ACTB	1.14	1.21	1.06	1.44	3.00
2	UBE2D2	1.23	1.35	1.28	1.49	4.00
3	RPS18	0.57	1.05	0.96	1.21	1.19
4	GAPDH	0.78	1.05	0.90	1.27	1.41

References

1.Dheda K, Huggett JF, Bustin SA, Johnson MA, Rook G, Zumla A. Validation of housekeeping genes for normalizing RNA expression in real-time PCR. *Biotechniques* (2004)37(1):112-114, doi: 10.2144/04371RR03.

2.Roy JG, McElhaney JE, Verschoor CP. Reliable reference genes for the quantification of mRNA in human T-cells and PBMCs stimulated with live influenza virus. *BMC Immunol* (2020) 21(1):4. doi: 10.1186/s12865-020-0334-8.
